# Measuring Patient Adherence to Malaria Treatment: A Comparison of Results from Self-Report and a Customised Electronic Monitoring Device

**DOI:** 10.1371/journal.pone.0134275

**Published:** 2015-07-27

**Authors:** Katia Bruxvoort, Charles Festo, Matthew Cairns, Admirabilis Kalolella, Frank Mayaya, S. Patrick Kachur, David Schellenberg, Catherine Goodman

**Affiliations:** 1 Department of Global Health and Development, London School of Hygiene and Tropical Medicine, London, United Kingdom; 2 Impact Evaluation Thematic Group, Ifakara Health Institute, Dar es Salaam, Tanzania; 3 Department of Infectious Disease Epidemiology, London School of Hygiene and Tropical Medicine, London, United Kingdom; 4 Malaria Branch, U.S. Centers for Disease Control and Prevention, Atlanta, United States of America; 5 Department of Disease Control, London School of Hygiene and Tropical Medicine, London, United Kingdom; Royal Tropical Institute, NETHERLANDS

## Abstract

**Background:**

Self-report is the most common and feasible method for assessing patient adherence to medication, but can be prone to recall bias and social desirability bias. Most studies assessing adherence to artemisinin-based combination therapies (ACTs) have relied on self-report. In this study, we use a novel customised electronic monitoring device—termed smart blister packs—to examine the validity of self-reported adherence to artemether-lumefantrine (AL) in southern Tanzania.

**Methods:**

Smart blister packs were designed to look identical to locally available AL blister packs and to record the date and time each tablet was removed from packaging. Patients obtaining AL at randomly selected health facilities and drug stores were followed up at home three days later and interviewed about each dose of AL taken. Blister packs were requested for pill count and extraction of smart blister pack data.

**Results:**

Data on adherence from both self-report verified by pill count and smart blister packs were available for 696 of 1,204 patients. There was no difference between methods in the proportion of patients assessed to have completed treatment (64% and 67%, respectively). However, the percentage taking the correct number of pills for each dose at the correct times (timely completion) was higher by self-report than smart blister packs (37% vs. 24%; p<0.0001). By smart blister packs, 64% of patients completing treatment did not take the correct number of pills per dose or did not take each dose at the correct time interval.

**Conclusion:**

Smart blister packs resulted in lower estimates of timely completion of AL and may be less prone to recall and social desirability bias. They may be useful when data on patterns of adherence are desirable to evaluate treatment outcomes. Improved methods of collecting self-reported data are needed to minimise bias and maximise comparability between studies.

## Introduction

Self-reported adherence, based on detailed questionnaires, is considered the most feasible method for assessing adherence in resource-poor settings [1, 2]. Self-reported data are relatively low cost to collect and do not involve complicated field logistics or invasive procedures such as blood sampling. However, despite its many advantages, self-reported adherence is prone to several sources of bias [2–4]. Data may be subject to recall bias if patients do not accurately recall their treatment history, including the number of pills taken, day and time of each dose, and when the full course was completed. Social desirability bias may also occur if patients provide perceived expected responses in order to avoid being seen as negligent.

Several other methods have been used to complement or even replace patient recall. Many studies verify self-reported adherence by counting the number of pills remaining in packaging, although this is not always accurate as patients may remove pills without taking them, or packaging may not be available for inspection [5, 6]. Electronic methods of assessing adherence have been used extensively in chronic diseases, mostly in the form of pill containers with caps that record the day and time the container is opened, such as Medication Events Monitoring Systems (MEMS) [4, 7]. While not feasible for routine clinical practice in many parts of the world, MEMS have been used to validate other adherence measures in studies of adherence to antimalarial drugs [8, 9], tuberculosis treatment [10], and antiretroviral therapy [11].

Artemisinin-based combination therapies (ACTs) are first line treatment for malaria in most endemic countries and are increasingly obtained by patients seeking treatment in both public and private health sectors. Good patient adherence is required to maximise their clinical impact and minimise the rate of development of drug-resistance [12–14]. A number of studies assessing adherence to ACTs have been conducted in recent years, with results showing that anywhere between 39% and 100% of patients can be considered adherent [5, 6], reflecting both genuine differences in adherence as well as variation in study design and measurement methods. The majority of these studies relied on self-report with or without pill count. One study in Malawi used MEMS containers and found 100% adherence to artemether-lumefantrine (AL) by self-report and lower adherence (92%) by MEMS [8]. However, ACTs are now typically dispensed in blister packs designed to improve adherence [15] and look considerably different than MEMS containers. This may result in over-estimated adherence, as patients using MEMS are likely to be aware that their adherence is being monitored and their experience is no longer comparable to that of patients receiving unit doses in their customary packaging.

In this study, set in southern Tanzania, the validity of self-reported patient adherence to AL was assessed using a novel customised electronic monitoring device—termed smart blister packs—that looked identical to regular AL packs, but contained a device that registered the date and time each pill was removed from the pack. This is the first study to our knowledge to employ this technology under routine conditions to investigate adherence to antimalarial treatment.

## Methods

### Study setting

The study was conducted in Mtwara, a rural region in southeastern Tanzania with more than a third of the population in the lowest national wealth quintile [16]. Community prevalence of falciparum malaria parasitaemia among children 6–59 months of age in Mtwara was 17.4% in the 2011–2012 HIV/AIDS and Malaria Indicator Survey [17].

In Tanzania, AL was introduced in public health facilities for treatment of uncomplicated malaria in 2006. The recommended treatment regimen is six doses over 3 days, with 1–4 tablets (each containing 20 mg artemether / 120 mg lumefantrine) per dose depending on the patient's weight / age band. National guidelines state that the second dose should be taken eight hours after the first dose, followed by the remaining doses each morning and evening of the second and third days [18].

In Tanzania’s private sector, more than two thirds of antimalarial drug sales occur in small drug stores [19], many of which have been upgraded to Accredited Drug Dispensing Outlets (ADDOs) through a process of training and accreditation. ADDOs are allowed to sell a limited number of prescription-only drugs, including some antibiotics and ACTs [20, 21]. By 2011, all drug shops in Mtwara were officially required to have upgraded to ADDO status, but in practice some shops had not yet paid fees or received training and were tolerated as “prospective ADDOs” (in this paper the term ADDOs is used to include both accredited outlets and “prospective ADDOs”).

This study was embedded in two parallel and contemporaneous studies of adherence to AL: a cluster randomised trial of a text message intervention targeted at ADDO dispensers to improve knowledge of advice to provide when dispensing AL, and an observational adherence study in public health facilities. Details and results of these studies are presented separately [22, 23]. Smart blister pack data were collected from a subset of patients in both studies for the analyses reported here.

### Smart blister packs

**Fig 1:**
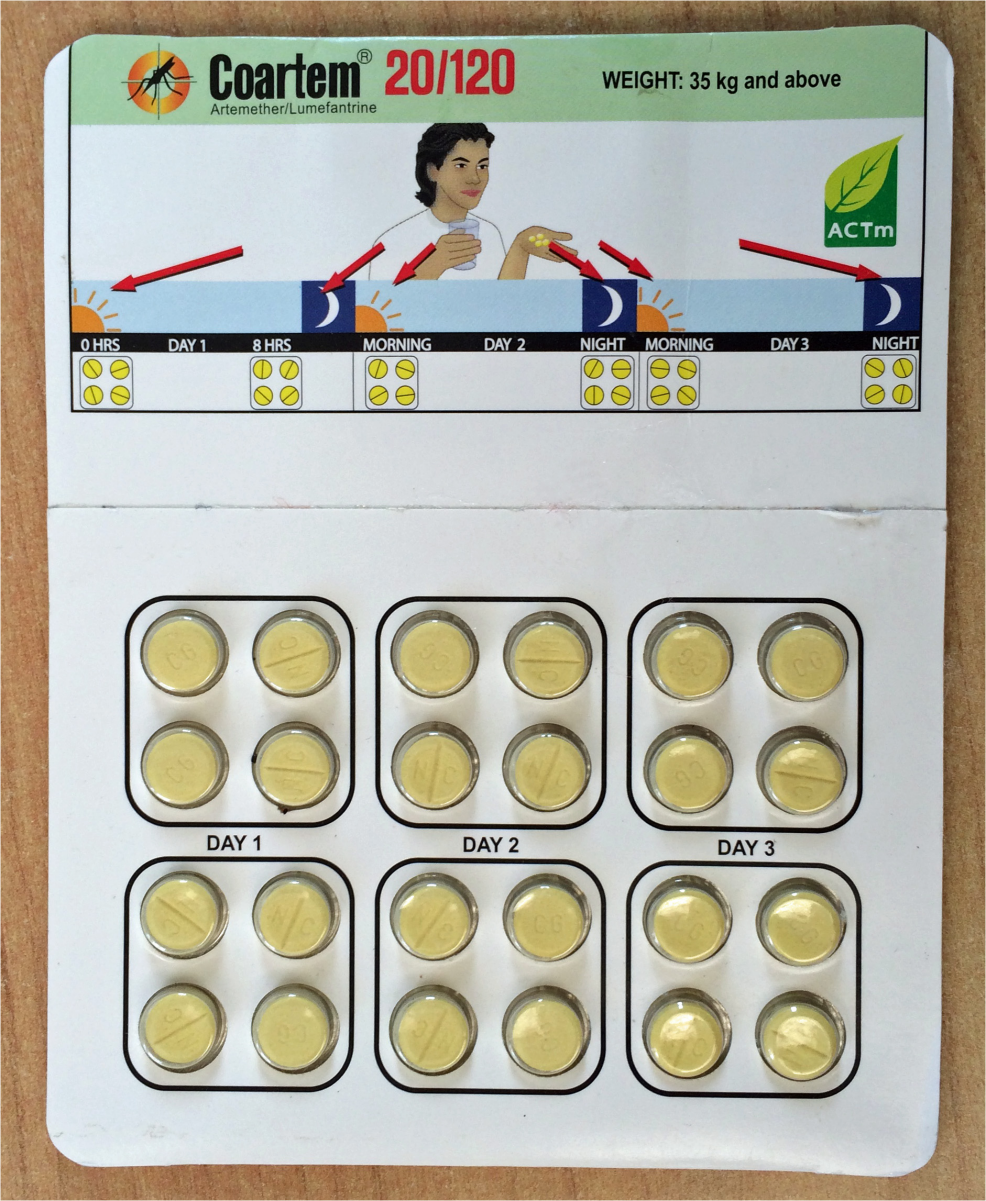
Picture of a smart blister pack showing resemblance to regular blister packs.

Smart blister packs were prepared using Med-ic blister package technology (Information Mediary Corporation, Ottawa, Canada). This comprises a fine wire, connected to a microchip, across each blister. When a tablet is pushed through the foil, the wire is disrupted, and the precise date and time this occurs is recorded on the chip. Wallet cards were designed for all four weight / age bands of AL (Coartem) and looked identical to AL locally available in Tanzania, but with a slight thickness near the top centre illustrations due to the electronic tag and cell battery (Fig 1). Smart blister packs were assembled by research assistants at the Ifakara Health Institute by folding the wallet cards around blister packs of Coartem purchased in Tanzania and sealing adhesively. The blister packs were activated prior to the study by scanning with a portable device developed by Information Mediary Corporation, allowing dates and times to be recorded. Following treatment, collected blister packs were scanned to retrieve data, and intervals between timestamps were calculated.

### Sample size and selection of study outlets

We aimed to provide smart blister packs to all 936 patients required for the cluster randomised trial in ADDOs [22] and all 448 patients required for the observational adherence study in public health facilities [23]. However, we also explored with what precision we could estimate sensitivity and specificity of self-report compared to smart blister packs. The calculations indicated that, assuming conservatively that sensitivity and specificity of self-report were 50%, with a true adherence of 60% and a design effect due to correlation of responses within outlets of 2.5, 600 patients with both self-report and smart blister pack data were sufficient to estimate sensitivity and specificity with a precision of 10 percentage points [24]. A small number of patients per outlet was desired in order to reduce any potential bias caused by increasing community awareness of the study's objectives. Thus, 40 public health facilities (but not hospitals) were randomly selected from a list of all dispensaries and health centres in Mtwara, and 82 ADDOs meeting study inclusion criteria [22] were randomly selected from a census register of all ADDOs in Mtwara.

### Study procedures

From September through November 2012, dispensers at selected public health facilities and ADDOs were visited by study supervisors and given a standard introduction about the study's objectives. Dispensers were provided with smart blister packs of AL to be dispensed in public health facilities to patients prescribed ACTs, and in ADDOs to patients indicating an intention to purchase treatment for malaria. In order to limit patients’ awareness of our primary interest in assessing adherence, which could have led to a biased assessment, dispensers were not told that the blister packs provided were any different than the regular AL packs used locally. Dispensers were told we would visit at home some, but not all, patients obtaining treatment for fever and were asked to fill out a registration form for all fever patients, including a description of where patients lived. Study staff visited outlets every day to check and collect registration forms. The intention was to register 12 patients obtaining ACTs in one week per outlet, but it took 2–3 weeks to recruit this number in some outlets.

Eligible patients who obtained AL were identified from the registration forms and followed up three days later (day 4). Where written informed consent was given, patients / caregivers were asked about demographic and socioeconomic characteristics, treatment-seeking history, symptoms, detailed information about each dose of AL taken, and advice provided by the dispenser. The blister packs were requested for a pill count and extraction of timestamp data, following a brief explanation of the nature and purpose of the smart blister packs. Blood samples were collected for a blood smear, a histidine-rich protein II (HRP-2)-based malaria rapid diagnostic test (mRDT) (Pf-specific from ICT Diagnostics, Cape Town, South Africa), and a filter paper dried blood spot (Whatman FTA DMPK B card, GE Healthcare). Results of blood smears and mRDTs are discussed in a previous paper [23]. Filter papers were transferred to the University Hospital in Lausanne, Switzerland, where they were analysed for blood concentration of lumefantrine by high performance liquid chromatography coupled to tandem mass spectrometry (LC-MS/MS). Due to technical problems that arose, these results are not shown, but the issue is addressed in the Discussion.

### Adherence definitions

Adherence was defined by both self-report and smart blister packs in two ways: verified completed treatment and verified timely completion [5]. Patients were considered to have verified completed treatment (hereafter termed completed treatment) by self-report if they reported taking all pills by the time of the follow up visit, verified by counting zero pills remaining in packaging. Patients who reported completing treatment but had pills remaining were considered non-adherent, as were patients who reported not completing treatment but presented an empty blister pack. Where blister packs were not available, self-report alone determined if patients completed treatment. By smart blister pack, patients were considered to have completed treatment if a timestamp was recorded for each pill by the time of the follow up visit.

Verified timely completion (hereafter termed timely completion) was a more stringent definition and included a time component. For self-report, Swahili times of day were used: “alfajiri” (early morning, defined here as 4:00 am–6:59 am), “asubuhi” (morning, 7:00 am–11:59 am), “mchana” (afternoon, 12:00 pm–3:59 pm), “jioni” (evening, 4:00 pm–6:59 pm), “usiku” (night, 7:00 pm–9:59 pm), and “usiku sana” (late night, 10:00 pm–3:59 am). Patients were considered to have self-reported timely completion if they took the correct number of pills for each dose and took each dose at the correct Swahili time of day. The second dose was considered correct if taken at the Swahili times of day corresponding with 8 hours after the beginning or end of the time interval for the Swahili time of day when the first dose was taken. For example, if the first dose was taken in the morning (“asubuhi”), then the second dose could be taken in the afternoon (“mchana”), evening (“jioni”), or night (“usiku”) of the same day (i.e. between 3:00 pm and 7:59 pm). Remaining doses were considered correct if taken at the Swahili times of day corresponding with 12 hours after the beginning or end of the time interval for the Swahili time of day when the previous dose was taken. As with completed treatment, examination of packaging when available was used to verify adherence.

By smart blister pack, patients were considered to have timely completion if they took the correct number of pills for each dose, and took the second dose eight hours plus or minus four hours after the first dose, followed by taking each of the remaining doses 12 hours plus or minus four hours after the previous dose.

For describing non-adherence, we distinguish between “intended doses” and “actual doses.” “Intended dose” refers to the pills that are intended by the manufacturer to be taken together at one of six specified times and, in the AL used in this study, grouped together in the blister packaging. “Actual dose” refers to pills that were actually taken together. An actual dose might have included pills that were not grouped together, or a different number than specified for the intended dose. Pills that were administered at least 30 minutes apart from each other were considered different actual doses.

### Data entry and analysis

All patient and dispenser interview data were collected using personal digital assistants, and data extracted from study forms (census, registration, and follow-up forms) were double entered into a Microsoft Access databases. Data were analysed in Stata 11.0 (Stata Corporation, College Station, USA). Confidence intervals for percentages were calculated using a linearized variance estimator, a robust standard error that accounts for the study design. McNemar’s analysis for paired data and conditional logistic regression were used to test the difference in completed treatment and timely completion between self-report and smart blister packs.

### Ethics

All questionnaires, consent forms, and other study documents were translated into Swahili and piloted prior to use. Written informed consent was collected from dispensers prior to census, patient registration, and interview and from patients or their caregivers prior to interview. The study protocol was approved by the ethical review boards of Ifakara Health Institute and London School of Hygiene and Tropical Medicine. CDC advisors provided technical assistance in design and analysis but were not engaged in data collection and did not have access to personal identifiers.

## Results

**Fig 2:**
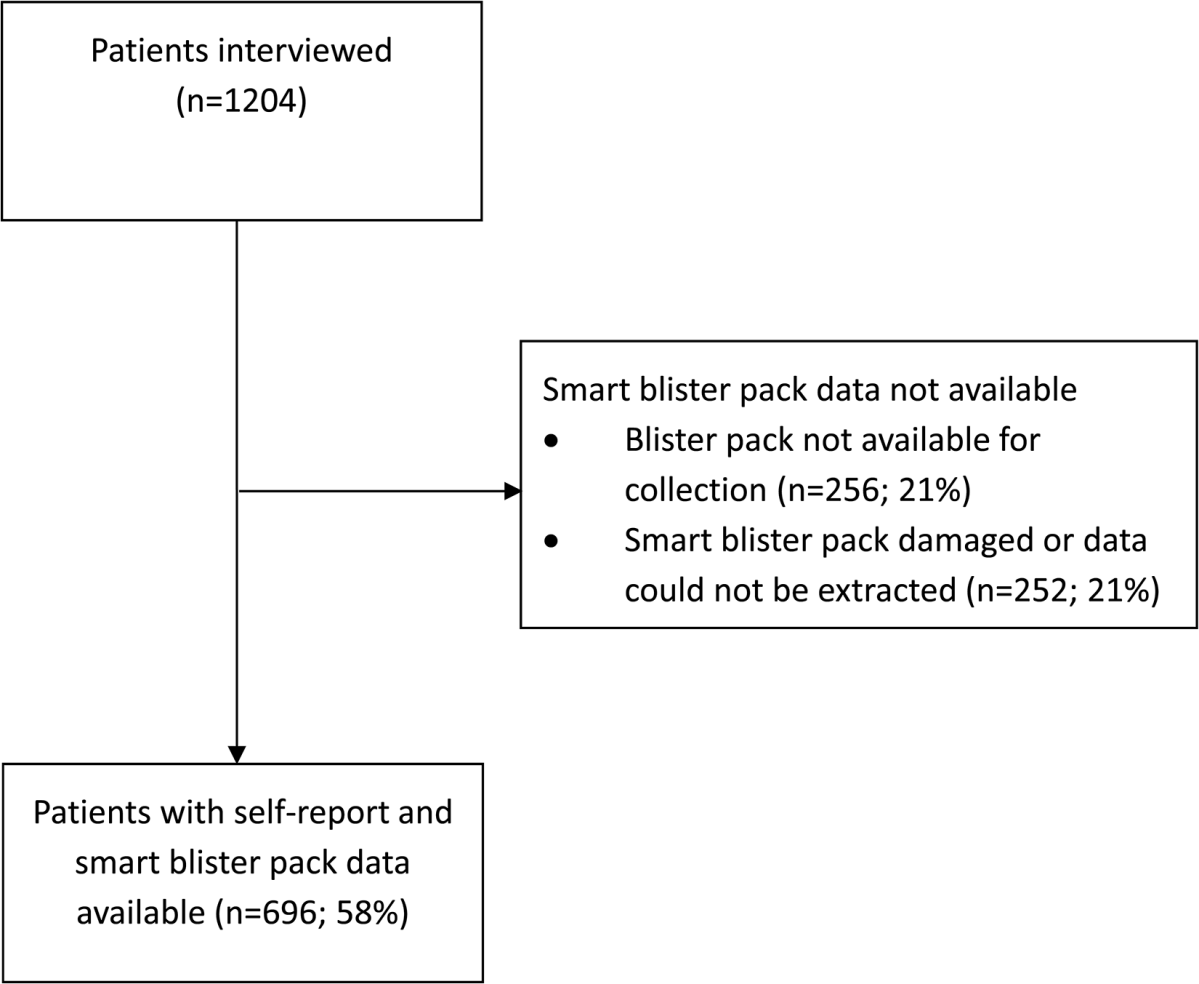
Flow chart of patients included in analysis.

Interviews were conducted with 1,204 patients from 117 outlets (five ADDOs were closed or refused to participate). Blister packs were not available for collection from 256 patients (21%). Smart blister packs that had been damaged or from which data were not extractable were collected from 252 patients (21%). In total, data were extracted from the blister packs of 696 patients (58%) (Fig 2). Due to infeasible dosing patterns or errors in smart blister pack technology, 55 patients were excluded from the analysis of timely completion, but not the analysis of completed treatment, since pills remained in their blister packs. For self-report, this applied to 18 patients who reported taking a subsequent dose before an earlier dose. For smart blister pack data, this applied to 10 patients with timestamps recorded before the treatment was dispensed or after the pack was collected, and 27 patients with a different number of pills observed by the study team at scanning than the number read by the software.

Table 1 shows characteristics of patients with both smart blister pack data and self-reported data available and patients with only self-reported data available (results comparing patient characteristics and adherence to AL across outlet types are presented elsewhere [21]). Based on the recommended age groups for AL blister packs in Tanzania, 27% of the 696 patients with both smart blister pack and self-reported data available were in the youngest age group (under three years), 24% were three years to under eight years, 6% were eight years to under 12 years, and 43% were 12 years and above. Fewer patients with only self-reported data available were under three years (21%) and more were 12 years and older (50%). Taking the first dose at the outlet was reported by 23% of patients with both smart blister pack and self-reported data available and 17% with only self-reported data available (p = 0.0281), but all other characteristics were similar for patients with and without both types of data. Given that some differences were observed, comparisons between methods were restricted to patients with both self-report and smart blister pack data available.

**Table 1:** Characteristics of patients with and without self-report and smart blister pack data available (percent (number))^1,2^.

	Patients with both smart blister pack and self-reported data available (N = 696)^1^	Patients with only self-reported data available (N = 508)^2^	p-value
Male	45.3 (315)	48.0 (244)	0.3
*Age* ^3^			0.0249
Under 3 years	26.9 (187)	21.1 (107)	
3 years to under 8 years	23.8 (166)	21.3 (108)	
8 years to under 12 years	5.8 (40)	8.1 (41)	
12 years and above	43.5 (303)	49.6 (252)	
Patient (or caregiver if patient below age 12 years) completed primary school	70.9 (490)	71.0 (360)	0.9
Slept under net the night before the interview	72.2 (502)	76.7 (388)	0.1
Attended an outlet in an urban ward	47.6 (331)	50.8 (258)	0.4
Attended ADDO (vs. public health facility)	64.2 (447)	66.9 (340)	0.2
Distance of 2.5 km or less from home to outlet (by GPS coordinates)	73.9 (468)	75.2 (340)	0.6
Reported being tested for malaria at outlet	26.4 (183)	24.2 (122)	0.5
Reported taking first dose of AL at the outlet	22.8 (158)	17.4 (88)	0.0281
Reported receiving correct instructions on AL regimen^4^	59.6 (281)	57.3 (291)	0.4

^1^ For patients with smart blister pack data and self-reported data available, data were missing for education for 5 patients, net use for 1 patient, GPS data for 63 patients, being tested for malaria for 2 patients, and taking the first dose of AL at the outlet for 4 patients.

^2^ For patients with only self-reported data available, data were missing for education for 3 patients, net use for 2 patients, GPS data for 73 patients, being tested for malaria for 5 patients, and taking the first dose of AL at the outlet for 2 patients.

^3^Age categories based on recommended age breakdown for AL blister packs in Tanzania.

^4^Reported the correct number of pills per dose, the correct number of doses per day, and the correct number of days per dispenser instructions.

### Comparison of adherence by self-report and smart blister pack data

#### Completed treatment

For patients with both types of data available, estimates of completed treatment were similar by smart blister packs (67%) and self-report (64%), with little difference in adherence by weight / age band of AL (Table 2). Considering smart blister packs as a gold standard, sensitivity and specificity of self-reported adherence were 96% and 100% respectively (S1 Table). It was not possible to calculate an odds ratio because there were no instances where patients who reported completing treatment did not complete treatment by smart blister pack data.

**Table 2:** Completed treatment by self-report and smart blister packs.

	Self-report	Smart blister packs
Number with self-reported data and electronic blister pack data available (N)	696	696
*Percent completed treatment (numerator / denominator) (95% CI)*		
**Total** ^**1**^	**64.1 (446/696) (59.8, 68.1)**	**66.7 (464/696) (62.3, 70.7)**
1x6 (6 tablets)	65.1 (162/249) (58.2, 71.4)	65.5 (163/249) (58.8, 71.6)
2x6 (12 tablets)	64.5 (80/124) (55.9, 72.3)	66.9 (83/124) (58.2, 74.6)
3x6 (18 tablets)	53.7 (22/41) (39.8, 67.0)	61.0 (25/41) (46.0, 74.1)
4x6 (24 tablets)	64.5 (182/282) (58.6, 70.1)	68.4 (193/282) (62.2, 74.1)

^1^An odds ratio for the effect of measurement method on completed treatment could not be calculated because there were zero patients who reported completing treatment but did not complete treatment by smart blister pack data.

Among patients who did not present blister packs for collection, self-reported completed treatment was higher (87%) compared to both all patients who presented blister packs (66%; p<0.0001) and patients for whom smart blister pack data were available (64%; p<0.0001). Self-reported completed treatment was slightly lower among patients with smart blister pack data available (64%) than among patients presenting damaged smart blister packs from which data were not extractable (72%; p = 0.050).

#### Timely completion

Timely completion was 37% by self-report and 24% by smart blister pack data (Table 3), much lower than completed treatment. The odds of timely completion by smart blister pack data were 0.36 times that of self-report (95% CI: 0.29, 0.43, p <0.0001). Sensitivity and specificity of self-reported timely completion were 74% and 74% respectively (S2 Table). Although the number of patients ages eight to 12 years with a 3x6 pack was relatively small, this group appeared to have slightly higher timely completion, especially by smart blister pack data (Table 3), than patients taking other packs. Adult patients (with 4x6 packs) also appeared to have slightly higher timely completion than the two youngest age groups according to smart blister pack data, but not self-report.

**Table 3:** Timely completion by self-report and smart blister packs^1^ (percent (number)).

	Self-report^1^		Smart blister packs^1^	
	Patients completing treatment	All patients	Patients completing treatment	All patients
**Total**	**418**	**641**	**436**	**641**
1x6 (6 tablets)	154	239	155	239
2x6 (12 tablets)	77	121	80	121
3x6 (18 tablets)	21	36	24	36
4x6 (24 tablets)	166	245	177	245
***Percent taking the correct number of pills for each of six actual doses*** ^***2***^ ^**,**^ ^***3***^				
**Total**	**96.9 (405)**	**63.5 (407)**	**67.2 (293)**	**45.7 (293)**
1x6 (6 tablets)	96.1 (148)	62.3 (149)	75.5 (117)	49.0 (117)
2x6 (12 tablets)	98.7 (76)	62.8 (76)	71.3 (57)	47.1 (57)
3x6 (18 tablets)	100 (21)	58.3 (21)	62.5 (15)	41.7 (15)
4x6 (24 tablets)	96.4 (160)	65.7 (161)	58.8 (104)	42.5 (104)
***Percent taking six actual doses at the correct time intervals*:** ^**4**^ ^**,**^ ^**5**^				
**Total**	**58.4 (244)**	**39.6 (254)**	**40.4 (176)**	**27.6 (177)**
1x6 (6 tablets)	57.8 (89)	38.1 (91)	32.9 (51)	21.3 (51)
2x6 (12 tablets)	58.4 (45)	38.0 (46)	36.3 (29)	24.8 (30)
3x6 (18 tablets)	71.4 (15)	44.4 (16)	54.2 (13)	36.1 (13)
4x6 (24 tablets)	57.2 (95)	41.2 (101)	46.9 (83)	33.9 (83)
***Timely completion (Percent taking the correct number of pills at the correct time intervals for each of six actual doses)*:**				
**Total** ^**6**^	**57.4 (240)**	**37.4 (240)**	**35.6 (155)**	**24.2 (155)**
1x6 (6 tablets)	56.5 (87)	36.4 (87)	32.9 (51)	21.3 (51)
2x6 (12 tablets)	58.4 (45)	37.2 (45)	32.5 (26)	21.5 (26)
3x6 (18 tablets)	71.4 (15)	41.7 (15)	50.0 (12)	33.3 (12)
4x6 (24 tablets)	56.0 (93)	38.0 (93)	37.3 (66)	26.9 (66)

^1^55 patients were excluded from this analysis because data on timing of each actual dose were not possible to assess for self-report for 18 patients and for smart blister pack data for 37 patients.

^2^“Actual doses” refers to pills actually taken together, including pills that were not grouped together, or a different number than specified for the intended dose. Pills administered at least 30 minutes apart from each other were considered different actual doses.

^3^ By self-report, number of pills taken missing for one dose for 2 patients for the 1x6 pack, 1 patient for the 2x6 pack, and 8 patients for the 4x6 pack.

^4^ By self-report, time of taking one or more doses missing for 24 patients for the 1x6 pack, 12 patients for the 2x6 pack, 2 patients for the 3x6 pack, and 20 patients for the 4x6 pack.

^5^By smart blister pack data, no patients who completed treatment and took more than six actual doses took the first six at the correct intervals.

^6^ For all patients (total columns) the odds ratio for timely completion by smart blister pack vs. self-report was 0.36, 95% CI: 0.29, 0.42; p<0.0001.

Timely completion consisted of two aspects, taking the correct number of pills for each dose and taking all six doses at the correct time intervals. Of the 418 patients with data on timely completion available who reported having completed treatment (Table 3), 97% reported taking the correct number of pills for each of six actual doses. In contrast, according to smart blister pack data, only 67% of the 436 patients completing treatment appeared to have taken the correct number of pills for each of six actual doses, a finding more pronounced for younger children than older children and adults. Of patients who reported completing treatment, 58% reported taking all six doses at the correct time intervals, compared with 40% of those completing treatment based on smart blister pack data. Thus, by self-report, correct time intervals were the primary obstacle for timely completion among patients who had completed treatment, but the smart blister pack data revealed problems both with the correct number of pills for each dose and the correct time intervals between doses.

### Patterns of non-adherence

**Fig 3:**
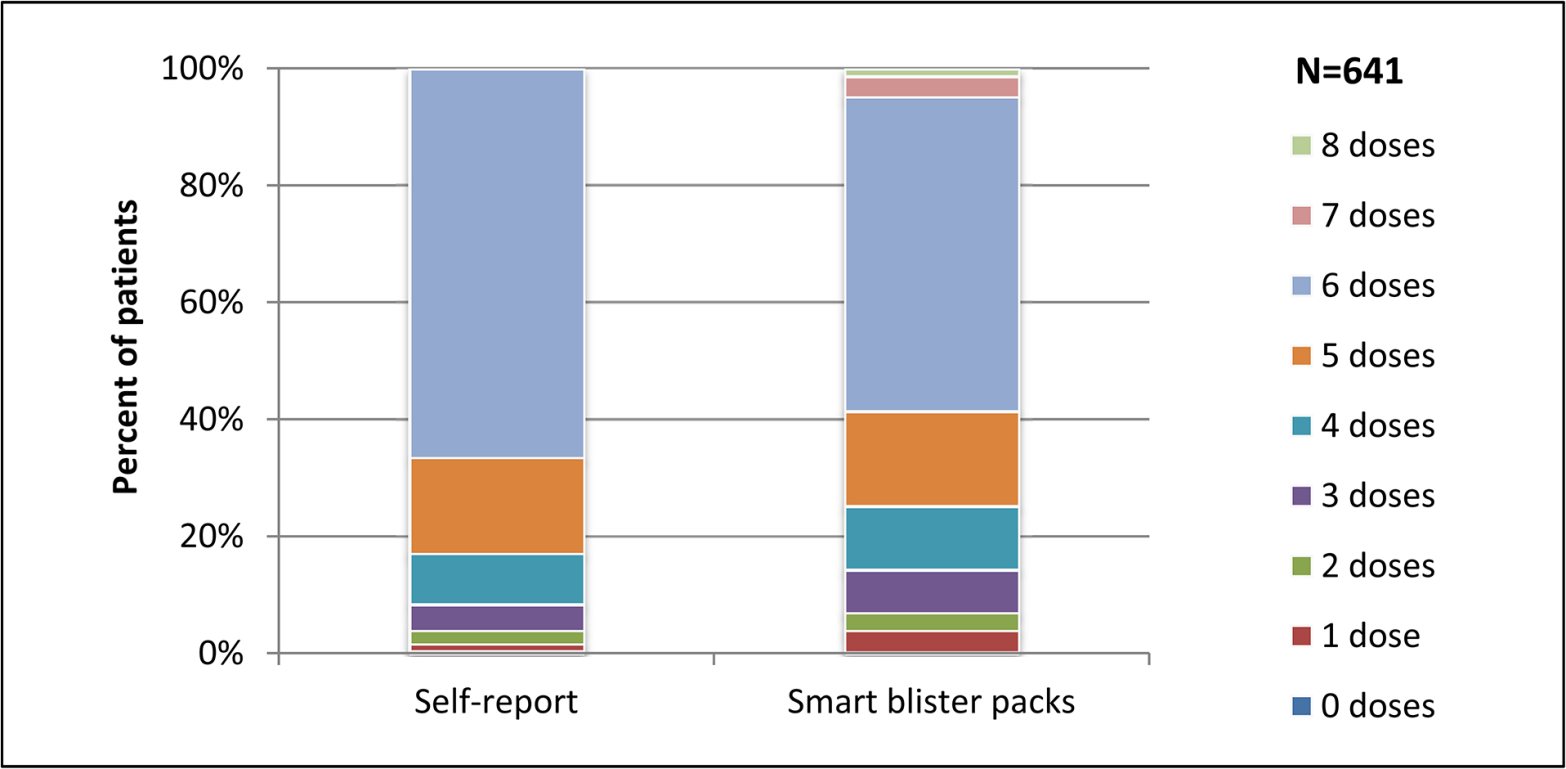
Number of actual doses taken by self-report and smart blister packs^1–3^. ^1^55 patients were excluded from this analysis because data on timing of each actual dose were not possible to assess for self-report for 18 patients and for smart blister pack data for 37 patients. ^2^“Actual doses” refers to pills actually taken together, including pills that were not grouped together, or a different number than specified for the intended dose. Pills administered at least 30 minutes apart from each other were considered different actual doses. ^3^By self-report, patients were asked only about each of the six intended doses.

Fig 3 shows the number of actual doses taken, ranging from 0–6 by self-report and from 0–8 by smart blister pack data. Overall, the proportions reporting completion of each dose were similar, except that by self-report more people reported taking six doses. This may be because patients were asked only about each of the six intended doses. Only about 5% of patients took more than six actual doses according to smart blister pack data, including both patients who completed treatment and those who did not (Fig 3 and Table 4). For both self-report and smart blister pack data, the median numbers of actual doses taken by patients who did not complete treatment were 4 for the 1x6 and 2x6 packs and 5 for the 3x6 and 4x6 packs. By self-report and smart blister pack data, respectively, the median total numbers of pills taken for patients who did not complete treatment were 4 and 4.5 for the 1x6 pack, 8 (by both methods) for the 2x6 pack, 15 and 13.5 for the 3x6 pack, and 20 (by both methods) for the 4x6 pack (Table 4).

**Table 4:** Median number of actual doses and pills taken by self-report and smart blister packs ^1^.

	Self-report^1^			Smart blister packs^1^		
	Patients completing treatment	Patients not completing treatment	All patients	Patients completing treatment	Patients not completing treatment	All patients
**Total**	**418**	**223**	**641**	**436**	**205**	**641**
1x6 (6 tablets)	154	85	239	155	84	239
2x6 (12 tablets)	77	44	121	80	41	121
3x6 (18 tablets)	21	15	36	24	12	36
4x6 (24 tablets)	166	79	245	177	68	245
***Median (range) number of actual doses taken*** ^***2***^						
**Total**	**6 (6–6)**	**5 (0–6)**	**6 (0–6)**	**6 (1–8)**	**4 (0–7)**	**6 (0–8)**
1x6 (6 tablets)	6 (6–6)	4 (1–5)	6 (1–6)	6 (1–6)	4 (1–5)	5 (1–6)
2x6 (12 tablets)	6 (6–6)	4 (0–5)	6 (0–6)	6 (1–7)	4 (1–7)	6 (1–7)
3x6 (18 tablets)	6 (6–6)	5 (2–6)	6 (2–6)	6 (1–8)	5 (2–6)	6 (1–8)
4x6 (24 tablets)	6 (6–6)	5 (0–6)	6 (0–6)	6 (1–8)	5 (0–7)	6 (0–8)
***Median (range) number of pills taken*** ^***3***^ ^**,**^ ^***4***^						
1x6 (6 tablets)	6 (6–7)	4 (1–6)	6 (1–7)	6 (6–6)	4.5 (1–5)	6 (1–6)
2x6 (12 tablets)	12 (12–13)	8 (0–12)	12 (0–13)	12 (12–12)	8 (1–11)	12 (1–12)
3x6 (18 tablets)	18 (18–18)	15 (6–15)	18 (6–18)	18 (18–18)	13.5 (6–15)	18 (6–18)
4x6 (24 tablets)	24 (24–24)	20 (0–24)	24 (0–24	24 (24–24)	20 (0–22)	24 (0–24)

^1^55 patients were excluded from this analysis because data on timing of each actual dose were not possible to assess for self-report for 18 patients and for smart blister pack data for 37 patients.

^2^“Actual doses” refers to pills actually taken together, including pills that were not grouped together, or a different number than specified for the intended dose. Pills administered at least 30 minutes apart from each other were considered different actual doses.

^3^By self-report, number of pills taken was missing for one dose for 2 patients for the 1x6 blister pack, 1 patient for the 2x6 blister pack, and 8 patients for the 4x6 blister pack.

^4^6 patients reported taking all pills, but since pills remained in the blister pack, they were not considered to have completed treatment (4 patients taking the 1x6 pack, 1 patient for the 2x6 pack, and 1 patient for the 4x6 pack). 1 patient for the 2x6 pack and 2 patients for the 4x6 blister reported taking no actual doses.

**Fig 4:**
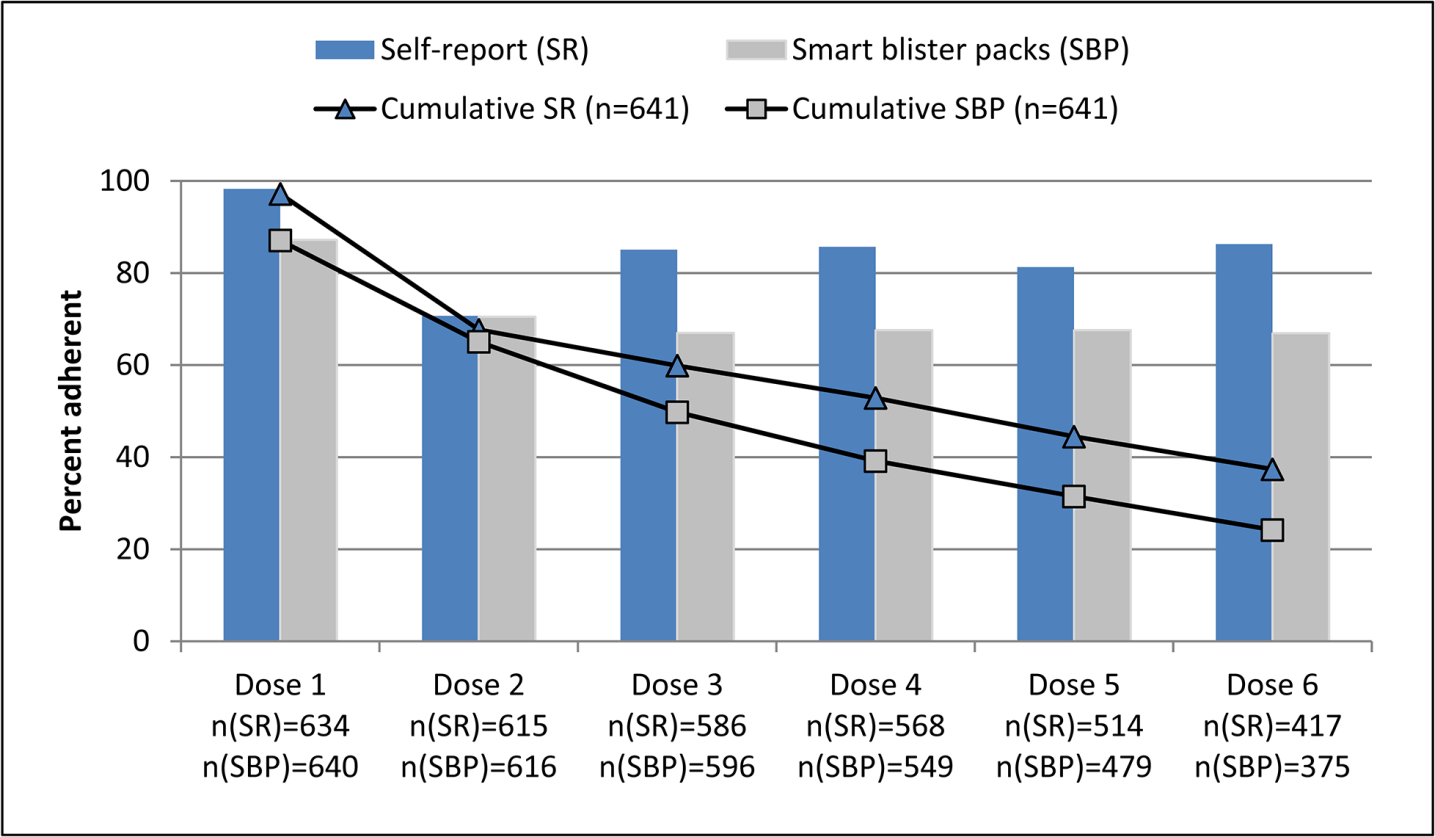
Timely completion for each actual dose and cumulatively^1^. ^1^55 patients were excluded from this analysis because data on timing of each actual dose were not possible to assess for self-report for 18 patients and for smart blister pack data for 37 patients.

For the first actual dose, timely completion (based only on taking the correct number of pills for each dose, as timeliness of the first dose was not assessed) was 98% by self-report and 87% by smart blister pack data. Timely completion for the second actual dose decreased to 71% by both self-report and smart blister pack data (Fig 4). For the third dose onward, timely completion by self-report was clearly higher than for the second dose (above 80%), but this increase was not evident in the smart blister pack data. Cumulatively, timely completion after two actual doses was similar for self-report and smart blister pack data, reflecting a slightly larger drop in timely completion by self-report. From dose three onwards, timely completion declined more rapidly by smart blister pack data.

## Discussion

This paper examines the validity of self-reported adherence in comparison with smart blister packs that recorded the day and time pills were removed from packaging. Timely completion (37% by self-report and 24% by smart blister packs) was much lower than completed treatment (64% by self-report and 67% by smart blister packs). No difference was observed between self-report and smart blister pack data for the percentage of patients completing treatment, but timely completion was lower when assessed using smart blister pack data (OR = 0.36, 95% CI: 0.29, 0.42, p<0.0001). Smart blister pack data showed that, even among patients who completed treatment, 33% did not take the correct number of pills for all doses, and 60% did not take each dose at the correct time interval.

This study has several limitations. First, recovery of blister packs might have been higher had dispensers and patients been more aware of the aims of the study. However, to avoid artificially increasing adherence, we intentionally did not tell dispensers or patients that the objective of the study was to assess adherence. Secondly, dispensers did not always accurately record the time that drugs were dispensed, despite daily visits by the study team and attempts to clarify times that were unclear. We therefore concluded that the data were not sufficiently robust to assess the timeliness of the first dose.

For both self-report and smart blister pack data, we allowed a margin of error around the correct time intervals when assessing timely completion. Clocks are not commonly used in rural Mtwara, and Swahili times of day which each cover several hours were therefore used for self-reported timely completion. Recorded timestamps plus or minus four hours were used to define acceptable limits for smart blister pack data. Among patients with timely completion by both methods, Swahili times of day corresponded well with timestamps, so it is unlikely that the definitions of timely completion affected the difference between methods. However, more stringent definitions for both methods might have resulted in lower levels of timely completion. Variability in adherence definitions has been frequently noted as a challenge for comparing adherence results across studies [5, 6, 25].

Self-report and smart blister pack data have various advantages and disadvantages for assessing patient adherence to ACTs. Self-reported adherence is collected through interviews, which are relatively inexpensive and are a widely accepted method of collecting data in many populations, including in southern Tanzania. Nonetheless, collecting accurate self-reported data can be challenging. Patients may have provided expected answers on the number of pills taken for each dose, or not remembered, and interviewers reported that patients often seemed confused about when and how each dose was taken. Some patients reporting taking more pills in total than the number in the blister pack, and some reported taking a subsequent dose before a previous dose. In this study, specific questions were asked about each of the six intended doses. However, patients may have taken pills grouped in a way that differed from the intended doses in the blister packs, and may therefore have taken more than six actual doses, as smart blister pack data indicated was the case. If any actual doses beyond the six intended are not captured by self-report interviews, this could underestimate the percentage completing treatment. In order to avoid some of these challenges, data collection tools need to be improved and evaluated, for example, including both open and closed-ended questions to identify actual doses taken [6].

As part of both the completed treatment and timely completion definitions, self-report was verified by counting pills remaining in packaging for the 83% of patients that presented blister packs. Very few patients (9/696) reported completing treatment but had pills remaining, similar to the high concordance between self-report alone and pill count described in a review of adherence to medication across diseases [3]. While this suggests that definitions incorporating pill counts may not differ from self-report alone, the patients who did not present blister packs reported higher completed treatment than those who did present blister packs (85% vs. 68%). This suggests that requesting blister packs may reduce over-reporting of adherence, unless patients for whom blister packs were not available were more likely to have finished packs and disposed of them. Blister packs were requested at the end of the questionnaire, which means patients could have removed pills from the pack after completing the interview but prior to presenting the pack.

Compared to smart blister packs, self-report generally over-reported timely completion, consistent with most other studies of electronic monitoring of adherence across diseases [4]. However, 27% (41/155) of patients with timely completion by smart blister pack data did not report timely completion (S2 Table). This is likely due to patient confusion when reporting dose history, which was noted frequently by study staff, or possibly removing pills for later consumption. Studies of adherence to tuberculosis treatment and antiretroviral therapy have also documented that some patients non-adherent by self-report were adherent by electronic pill containers [10, 11]. Our smart blister pack data revealed how some patients took pills in more than six actual doses, or removed a large number of pills at once, including removal of all pills remaining in the packaging on the date of the interview—possibly at the time of the interview.

The smart blister packs were easy to assemble, the software and portable scanner were straightforward to use, and the packs were designed to look identical to regular AL packs commonly available in Tanzania. However, they had a slight bulge at the top of the package for a small chip that stored data. Some patients noticed this and opened up the packaging to investigate, damaging the capacity to record and extract timestamps. Several patients were alarmed when the chip was discovered, requiring the study team to provide full explanations to participants and village leaders. Other blister packs were destroyed when children were allowed to play with them after completion of treatment, or when dropped in water or fires before, during, or after taking pills.

Although smart blister packs accurately recorded when pills were removed from the packaging, it was also possible for a timestamp to be recorded for a pill if pressure had been applied and a pill was partially removed, even if the seal was not obviously broken. While it was evident when timestamps were recorded before the treatment was dispensed to the patient or after the pack was collected, this could also have occurred in the middle of treatment, which could have altered timely completion estimates, though this is not thought to have occurred frequently. In addition, for some patients, the number of pills observed by the study team at scanning did not correspond with the number read by the software. Future smart blister pack designs should be improved to reduce the chances that timestamps would be recorded when pills weren’t completely removed and to reduce the bulge in the packaging so that patients are less likely to notice and tamper with the packaging.

Another approach to getting insights into adherence is through the measurement of drug levels in patients’ blood during follow-up. In the case of ACTs, this has been approached by measuring the concentration of the non-artemisinin partner drug since the artemisinin component is absorbed and eliminated rapidly [26]. We collected blood spots on filter paper for the assessment of lumefantrine concentrations. However, analysis 19–24 months after collection found lumefantrine concentrations below the lower limit of quantification, reflecting the need for filter papers to be stored at appropriate temperatures and ideally analysed within 4–6 weeks post-collection [27].

Moreover, studies of adherence to AL have not found significant differences in blood lumefantrine concentrations between patients considered adherent and non-adherent by self-report [8, 28–30]. Some studies have reported significant differences in lumefantrine concentrations (usually measured 7 days after initiation of treatment) between supervised versus non-supervised patients [31–33]. These and other studies have not reported differences in treatment failure based on cut-offs of 280 ng / ml [29, 31–33] or 175 ng / ml [8], which have been previously found to predict recrudescence [12, 26]. In addition, lumefantrine is known to have high inter-individual metabolic variation, with factors such as weight, age, pregnancy, and fat intake affecting absorption [34–36]. As a result, while biological measures of lumefantrine concentration might provide some additional objective information about adherence, they can be difficult to obtain, process and interpret in follow-up studies.

Finally, methods for measuring adherence should be based on levels and components of adherence that are important for effectiveness. For example, it might be expected that completing treatment is more important than exact timing of doses. The recommended regimen for AL was developed in early trials showing that a six-dose regimen of AL taken twice per day, with the second dose taken after eight hours, resulted in higher cure rates than a four-dose regimen [34, 37, 38]. However, it is unclear how strictly dose intervals must be adhered to in order for treatment to be effective. Patients who obtain ACTs in the evening may be less likely to adhere to correct dose timing than patients who obtain ACTs in the morning, and it is unclear to what extent this matters. As adherence needs to be defined and measured depending on what is required for drug effectiveness, the importance of exact dose timing must be clarified.

## Conclusion

Accurate measurements of patient adherence are important for developing strategies to assure the effectiveness of ACTs. While self-reported data along with examination of packaging might be sufficient to assess completion of treatment, patient reports of timely completion appear to be affected by both recall and/or social desirability bias. Smart blister packs provided slightly lower, and potentially more accurate, estimates of the number of pills taken for each dose and the time intervals between doses. In settings where data on dose timing are considered important for clinical outcomes, smart blister packs may be a useful tool, though innovations to make them more robust and discreet would be useful. Improved methods for collecting self-reported data are also likely to enhance the accuracy of measured patient adherence to treatment. Finally, a clearer rationale for what is considered adequate adherence is required.

## Supporting Information

S1 Table Matrix of completing treatment showing sensitivity and specificity of self-report compared to smart blister pack data. (DOCX)

S2 Table Matrix of timely completion showing sensitivity and specificity of self-report compared to smart blister pack data. (DOCX)
