# Supplementary material for: Measuring Patient Adherence to Malaria Treatment: A Comparison of Results from Self-Report and a Customised Electronic Monitoring Device
Source: PLoS One. 2015 Jul 27;10(7):e0134275. doi: 10.1371/journal.pone.0134275 (PMC4516331; doi:10.1371/journal.pone.0134275)
Supplement: S1 Table — (DOCX) [file pone.0134275.s001.docx]

**S1 Table. Matrix of completing treatment showing sensitivity and specificity of self-report compared to smart blister pack data. (Percent (number) (95% CI)).**

|  |  | Smart blister packs | | |
| --- | --- | --- | --- | --- |
|  |  | Completed treatment | Did not complete treatment | Total |
| Self-report | Completed treatment | 96.1 (446) (93.6, 97.7) | 0 | 64.1 (446) (59.8, 68.1) |
|  | Did not complete treatment | 3.9 (18) (2.32, 6.42) | 100 (232) | 35.9 (250) (31.9, 40.2) |
|  | Total | 100 (464) | 100 (232) | 100 (696) |
