# Supplementary material for: Measuring Patient Adherence to Malaria Treatment: A Comparison of Results from Self-Report and a Customised Electronic Monitoring Device
Source: PLoS One. 2015 Jul 27;10(7):e0134275. doi: 10.1371/journal.pone.0134275 (PMC4516331; doi:10.1371/journal.pone.0134275)
Supplement: S2 Table — (DOCX) [file pone.0134275.s002.docx]

**S2 Table. Matrix of timely completion showing sensitivity and specificity of self-report compared to smart blister pack data ((Percent (number)(95% CI)).**

|  |  | Smart blister packs | | |
| --- | --- | --- | --- | --- |
|  |  | Timely completion | No timely completion | Total |
| Self-report | Timely completion | 73.5 (114) (66.1, 79.8) | 25.9 (126) (21.7, 30.6) | 37.4 (240) (32.8, 42.3) |
|  | No timely completion | 26.5 (41) (20.2, 33.9) | 74.1 (360) (69.4, 78.3) | 62.6 (401) |
|  | Total | 100 (155) | 100 (486) | 100 (641) |
